# Modeling and Trajectory Tracking Model Predictive Control Novel Method of AUV Based on CFD Data

**DOI:** 10.3390/s22114234

**Published:** 2022-06-01

**Authors:** Han Bao, Haitao Zhu

**Affiliations:** 1 College of Mechanical and Electrical Engineering, Harbin Engineering University, Harbin 150001, China; baohan@hrbeu.edu.cn; 2 Yantai Research Institute and Graduate School, Harbin Engineering University, Yantai 265501, China

**Keywords:** autonomous underwater vehicle, model predictive control, trajectory tracking, normal probability division, GA-ACO algorithm, hydrodynamic analysis

## Abstract

In this paper, a novel model predictive control (MPC) method based on the population normal probability division genetic algorithm and ant colony optimization (GA-ACO) method is proposed to optimally solve the problem of standard MPC with constraints that generally cannot yield global optimal solutions when using quadratic programming (QP). Combined with dynamic sliding mode control (SMC), this model is applied to the dynamic trajectory tracking control of autonomous underwater vehicles (AUVs). First, the computational fluid dynamics (CFD) simulation platform ANSYS Fluent is used to solve for the main hydrodynamic coefficients required to establish the AUV dynamic model. Then, the novel model predictive controller is used to obtain the desired velocity command of the AUV. To reduce the influence of external interference and realize accurate velocity tracking, dynamic SMC is used to obtain the control input command. In addition, stability analysis based on the Lyapunov method proves the asymptotic stability of the controller. Finally, the trajectory tracking performance of the AUV in an underwater, three-dimensional environment is verified by using the MATLAB/Simulink simulation platform. The results verify the effectiveness and robustness of the proposed control method.

## 1. Introduction

The autonomous underwater vehicle (AUV) is a type of underwater unmanned vehicle (UUV) that has become important to ocean exploration and as a development tool in which artificial intelligence and other advanced computing technologies are integrated [1,2]. Because AUVs operate without an umbilical cord, they are more flexible in underwater operations [3]. In the early stages of deployment, AUVs were mainly used for the development of offshore oil and gas facilities, etc., although later, this technology played an important role in the field of military salvage [4,5]. The AUV has increasingly become of interest to researchers in the marine, oil, industry and military fields of countries around the world [6,7]. So that the AUV can successfully complete its assigned underwater operations, the performance of its control system is critical, especially the design of its trajectory tracking control method [8,9].

Trajectory tracking is limited by time constraints [10]. This technology feature only needs to track the reference path within a certain error allowable range to guide the AUV from the initial state to the final state [11]. For the state error of the system to converge to zero, the selection and optimization of the control method is highly important [11]. At present, many control methods have been successfully applied in the field of AUV trajectory tracking control, including proportional-integral-derivative (PID) control, neural network (NN) control, sliding mode control (SMC) and model predictive control (MPC) [12,13].

PID control is the most widely used control method in engineering, as well as the most stable. Among the various methods, the proportional (P) aspect corrects the deviation so that the process can respond quickly [14,15]. The integral (I) represents information accumulated in the past, which eliminates static errors and improves the static characteristics of the system [16]. The derivative (D) plays a leading control role in signal changes, reduces overshoot, overcomes oscillations and improves system stability [17]. However, an AUV is a high-inertia system. When the expected control command changes suddenly, the output of the AUV cannot be adjusted in time, which results in large system errors [18]. Therefore, PID control is usually combined with adaptive control, fuzzy control or neural network control, among others [19].

NN control is a current frontier in the field of automatic control [20,21]. This method represents a new branch of intelligent control, offering a new way to solve the control problems of complex nonlinear, uncertain and uncertain systems [22,23]. The NN controller is actually a feedforward controller that establishes the inverse model of the controlled object and adjusts the weights between the NN by learning the input and output of the traditional controller as samples to allow the feedback control input to approach zero and replace the traditional controller [24]. The advantage of NN control is that a precise dynamic model of the control target is not needed; additionally, NN can be used to approximate the nonlinear system [25]. The disadvantage is that it is difficult to obtain learning samples. Because the AUV system is highly nonlinear and strongly coupled, these uncertainties challenge the ability of the offline neural network controller in regard to robustness; as a result, the development of an online NN controller should be emphasized [26].

SMC is a special kind of nonlinear control that can, in essence, overcome the uncertainty of the system and has strong robustness in the face of an unknown disturbance, thus it is suitable for the control of AUVs [27]. However, when the state trajectory of the SMC reaches the sliding mode surface, it is difficult to slide strictly along the sliding mode toward the equilibrium point and instead passes back and forth on both sides of the SMC to approach the equilibrium point, resulting in chattering [28]. Elmokadem et al. derived an SMC method for the trajectory tracking control of an AUV but did not solve the chattering problem exhibited by the controller [29]. Londhe and Patre proposed an adaptive fuzzy sliding mode controller by deriving fuzzy logic rules using the Lyapunov energy function to reduce the SMC chattering problem [30].

When using the MPC feedback control method, the current control action is obtained by solving a finite time-domain open-loop optimal control problem at each sampling moment [31,32]. The current state of the process is the initial state of the optimal control problem, and the optimal control sequence only implements the first control effect [33,34]. The advantage of MPC is that it is good at dealing with problems with constraints. In traditional MPC, the process of solving the optimal control sequence is usually transformed into a quadratic programming (QP) problem, which solves the minimum value of a positive definite quadratic function with linear equality constraints [35,36]. However, the QP process often cannot obtain the global optimal solution. To solve this problem, the improved particle swarm optimization (PSO) algorithm, genetic algorithm (GA) and ant colony optimization (ACO) algorithm are usually introduced into MPC to replace the QP solution process. Ref. [37] Gan et al. applied quantum particle swarm optimization (QPSO) to model a predictive controller to solve the optimal control quantity. The application of QPSO enhanced the global search ability of traditional PSO and applied the controller to the trajectory tracking control field of AUVs, achieving a good tracking effect. Ref. [38] Zhang et al. proposed an improved GA to optimize the Q and R matrices in the control objective function of the MPC method; unfortunately, the GA easily falls into a local optimal value due to the lack of population diversity. Additionally, it is difficult for the algorithm to converge quickly in the later stage, and it is difficult to obtain the optimal solution within the MPC sampling time. The ACO algorithm can compensate for the slow convergence rate of the GA in the later stage, although the initial convergence rate of the algorithm is slow due to its low initial pheromone concentration.

To solve the above problems, this paper proposes an MPC method optimized by the GA-ACO algorithm based on population normal probability division. The standard GA of the fitness value according to the normal probability interval is divided into the following steps: put forward the internal and external hybrid crossover operator and dynamic hybrid mutation operator to maintain the diversity of the population, and later, using the ACO algorithm to accelerate convergence, make MPC obtain the global optimal control sequence. Finally, combine the process with dynamic SMC, which is used in AUV trajectory tracking control. The controller recalculates the optimal input at the next time according to the systematic error at each sampling time. The hydrodynamic coefficients used in AUV modeling in this paper are computational fluid dynamics (CFD) data based on the actual solution of ANSYS Fluent.

The main content of this paper is as follows: Section 2 introduces the kinematic and dynamical model of the AUV. In Section 3, the solution methods and results for the main hydrodynamic coefficients of the AUV are introduced. Section 4 introduces the formulation of the optimization problem and the control method proposed in this article. Section 5 presents a stability analysis of the proposed control method based on terminal constraints. Section 6 gives the simulation results. Section 7 offers some concluding comments.

## 2. Modeling of the AUV

**Figure 1:**
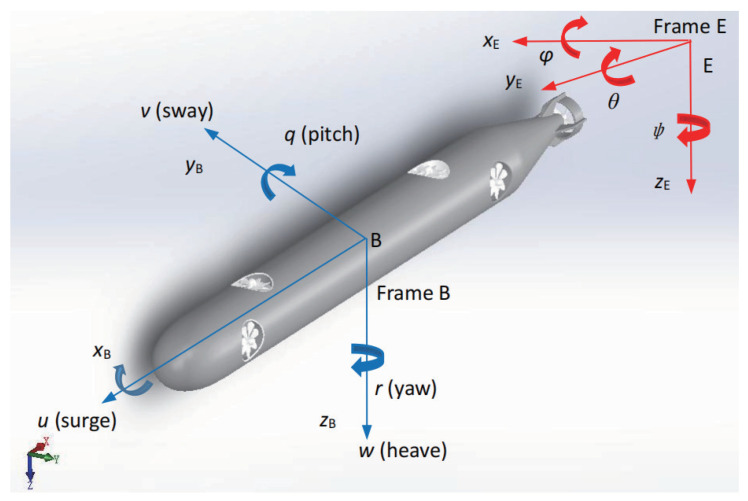
Reference frames.

This paper focuses on AUV modeling and trajectory tracking control. As shown in Figure 1, the size and mechanical structure of the AUV proposed in this paper are designed using the SOLIDWORKS design platform, with the relevant parameter values shown in Table 1. The arrangement of thrusters supports the movement of the AUV with five degrees of freedom. To avoid the singularity of the Euler angle, the pitch angle θ of the AUV is bounded and satisfies −π/2≤θ≤π/2. The AUV roll angle ϕ is also self-stable due to the recovery torque caused by buoyancy and other factors. Therefore, the default expected angle and velocity values of ϕ and θ in this paper are 0. In the forward direction, one main propeller is used to generate the thrust of the surge, two vertical propellers complete the thrust of the heave and the pitch moment, and two lateral propellers complete the thrust of the sway and the yaw moment.

### 2.1. Kinematic Modeling

To establish the kinematic model of the AUV to study its trajectory tracking control, the body coordinate frame *B* and the inertial coordinate frame *I* are introduced, both of which are defined in right-handed Cartesian coordinate systems. The origin of *B* is the center of gravity of the AUV, which is used to describe the velocity information of the AUV. The velocity vector of the AUV is defined as $v={\left[u,v,w,q,r\right]}^{T}$, where *u*, *v* and *w* are the velocities in the surge, sway and heave directions, respectively, and *q* and *r* are the angular velocities in the pitch and yaw directions. The origin of *I* may be any point on Earth, which is used to define the position and attitude information of the AUV in *B* with respect to *I*. The position and attitude vector of the AUV is defined as $\eta ={\left[x,y,z,\theta ,\psi \right]}^{T}$, where the positive direction of the *x*, *y* and *z* axes points in the horizontal north direction, the horizontal east direction and the geocentric direction, respectively, and θ and ψ are the pitch and yaw angle attitudes of the AUV.

The kinematic model can be defined in the following form:

(1) $$\dot{\eta }=J\left(\eta \right)v$$

Here, J(η) is the transition matrix between the two coordinate frames:

(2) $$J\left(\eta \right)=\left[\begin{array}{ccccc} \cos\theta \cos\psi & -\sin\psi & \sin\theta \cos\psi & 0 & 0\\ \cos\theta \sin\psi & \cos\psi & \sin\theta \sin\psi & 0 & 0\\ -\sin\theta & 0 & \cos\theta & 0 & 0\\ 0 & 0 & 0 & 1 & 0\\ 0 & 0 & 0 & 0 & 1/\cos\theta \end{array}\right]$$

### 2.2. Dynamic Modeling

The dynamic model of the AUV, as proposed by Fossen, is expressed [35]:

(3) $$M\dot{v}+C\left(v\right)v+D\left(v\right)v+g\left(\eta \right)=\tau$$

Here, $M=M_{\mathrm{RB}}+M_{A}$ is defined as the inertia matrix. $M_{\mathrm{RB}}=diag\left(m,m,m,I_{y},I_{z}\right)$ is the rigid-body inertial mass matrix, which is a property of the AUV itself and does not change with time, where *m* is the mass of the AUV and $I_{y}$ and $I_{z}$ are the inertia tensors of the $y_{B}$ and $z_{B}$ axes. $M_{A}=diag\left(-X_{\dot{u}},-Y_{\dot{v}},-Z_{\dot{w}},-M_{\dot{q}},-N_{\dot{r}}\right)$ is the additional mass matrix, which represents the hydrodynamic force due to the acceleration of the AUV underwater, where $-X_{\dot{u}}$, $-Y_{\dot{v}}$, $-Z_{\dot{w}}$, $-M_{\dot{q}}$ and $-N_{\dot{r}}$ are the inertial hydrodynamic coefficients.

$C\left(v\right)=C_{\mathrm{RB}}+C_{A}$ is the Coriolis and centripetal force matrix. $C_{\mathrm{RB}}$ is the Coriolis and centripetal force matrix due to the rigid-body inertial mass, and $C_{A}$ is the hydrodynamic Coriolis and centripetal force matrix due to the additional mass.

Specifically:

(4) $$C_{\mathrm{RB}}=\left[\begin{array}{ccccc} 0 & 0 & 0 & mw & -mv\\ 0 & 0 & 0 & 0 & mu\\ 0 & 0 & 0 & -mu & 0\\ -mw & 0 & mu & 0 & 0\\ mv & -mu & 0 & 0 & 0 \end{array}\right]$$

(5) $$C_{A}=\left[\begin{array}{ccccc} 0 & 0 & 0 & -Z_{\dot{w}}w & Y_{\dot{v}}v\\ 0 & 0 & 0 & 0 & -X_{\dot{u}}u\\ 0 & 0 & 0 & X_{\dot{u}}u & 0\\ Z_{\dot{w}}w & 0 & -X_{\dot{u}}u & 0 & 0\\ -Y_{\dot{v}}v & X_{\dot{u}}u & 0 & 0 & 0 \end{array}\right]$$

$D\left(v\right)=D_{L}\left(v\right)+D_{N}\left(v\right)$ is the fluid damping matrix, including the linear damping matrix $D_{L}\left(v\right)$ and the quadratic damping matrix $D_{N}\left(v\right)$, where $D_{L}\left(v\right)=diag(X_{u},Y_{v},Z_{w},M_{q},$$N_{r})$ and $D_{N}\left(v\right)=diag(X_{\left|u\right|u}\left|u\right|,Y_{\left|v\right|v}\left|v\right|,Z_{\left|w\right|w}\left|w\right|,M_{\left|q\right|q}\left|q\right|,$$N_{\left|r\right|r}\left|r\right|)$. Here, $X_{u}$, $Y_{v}$, $Z_{w}$, $N_{r}$, $X_{\left|u\right|u}$, $Y_{\left|v\right|v}$, $Z_{\left|w\right|w}$, $M_{\left|q\right|q}\left|q\right|$ and $N_{\left|r\right|r}$ are measurable viscous hydrodynamic coefficients.

$g\left(\eta \right)={\left[0,0,0,bZ_{g}\sin\theta ,0\right]}^{T}$ is a vector representing the restoring forces and moments due to the forces of gravity and buoyancy, *b*. In this paper, the force of gravity of the AUV is set equal to its buoyancy. $Z_{g}$ is the vertical coordinate of the center of gravity.

$\tau ={\left[\tau_{u},\tau_{v},\tau_{w},\tau_{q},\tau_{r}\right]}^{T}$ is the vector of the input forces and moments for each DOF.

## 3. Solution for the Hydrodynamic Coefficients

### 3.1. Solution Method and Fluid Area Division

**Figure 2:**
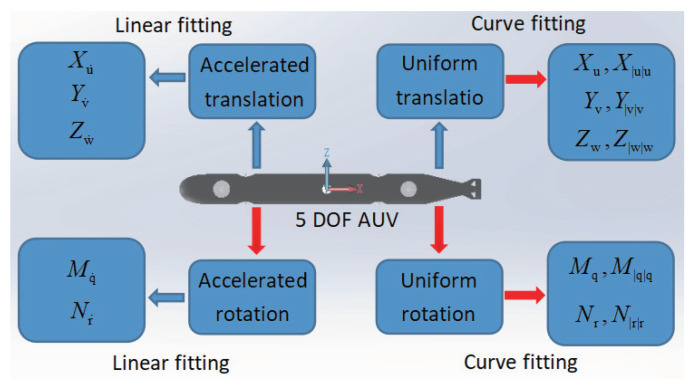
Solving method.

**Figure 3:**
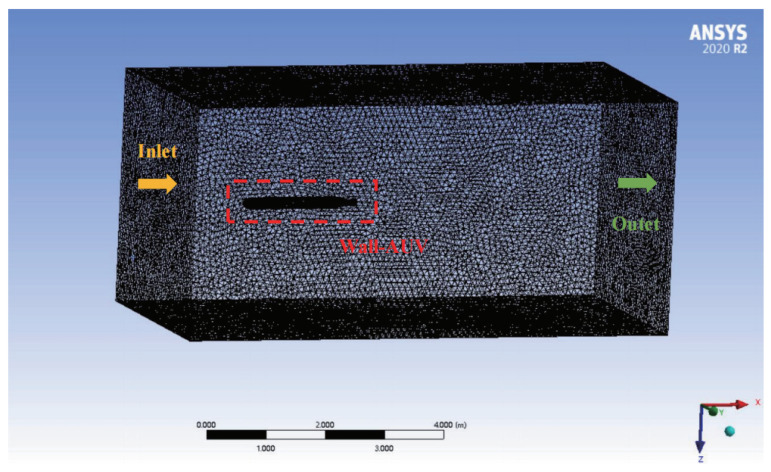
Solution area settings of translational motion.

**Figure 4:**
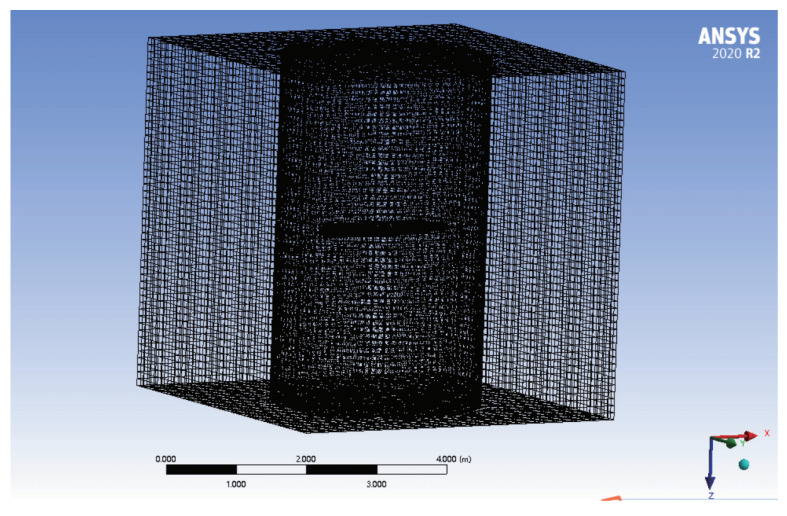
Solution area settings of rotation motion.

To realize the accurate modeling of the AUV as proposed in this paper, it is necessary to analyze its hydrodynamic performance and solve the main viscous hydrodynamic parameters and inertial hydrodynamic parameters. This part is based on the solution method of [39]. The solution process of this method is shown in Figure 2. Using the CFD solution ANSYS Fluent platform, the AUV simulates the corresponding translational motion and rotational motion in the seawater environment. Figure 3 shows the division of the solution domain for the translational motion, and Figure 4 shows the solution domain division of the rotational motion. The control method and parameter settings of the mesh divisions are shown in Table 2.

### 3.2. Simulation Process

As shown in Equation (3), through the analysis of each component in the dynamic model of the AUV, it can be concluded that the hydrodynamic force of the AUV when moving in the fluid includes the inertial hydrodynamic force or moment $\tau_{A}$ caused by the additional mass and the viscous drag or moment $\tau_{D}$ caused by the viscosity of the fluid. The expression is as follows:

(6) $$\tau_{D}=D_{L}\left(v\right)v+D_{N}\left(v\right)\left|v\right|v$$

(7) $$\tau_{A}=M_{A}\dot{v}$$

$D_{L}$ is the linear damping matrix, and $D_{N}$ is the nonlinear damping matrix. From Equation (6), it can be concluded that the viscous drag and moment of the AUV are related to velocity via a quadratic function. By simulating the translational and rotational motion of each degree of freedom of the AUV under different velocities, the values of drag and moment under different velocities can be solved, and the viscous hydrodynamic coefficients can be identified by curve fitting with the least squares method.

In all simulations, the fluid is seawater with a density of 1025 $\mathrm{kg}/m^{3}$ and viscosity of $8.8871\times 10^{-4}\;\mathrm{Pa}\cdot s$. The turbulence model is K-epsilon.

The uniform translational and rotational motions of the AUV are simulated. Each simulation only gives the velocity value of one degree of freedom to the AUV, while the velocity value of any other degrees of freedom is zero.

**Figure 5:**
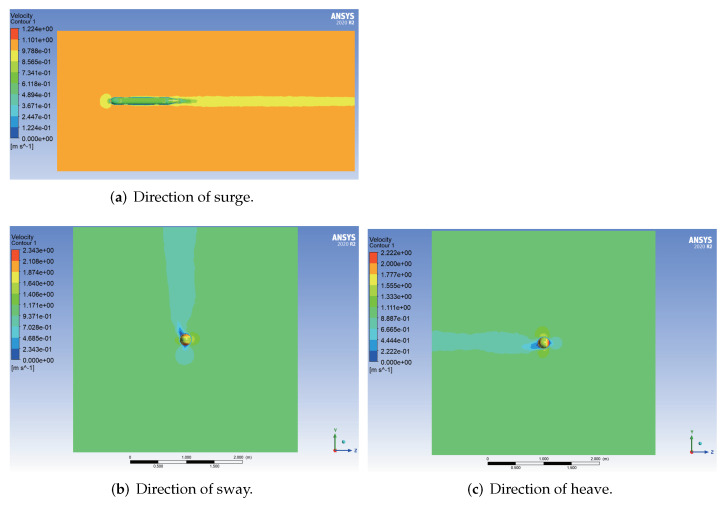
Velocity cloud diagrams of the AUV in uniform translational motion.

**Figure 6:**
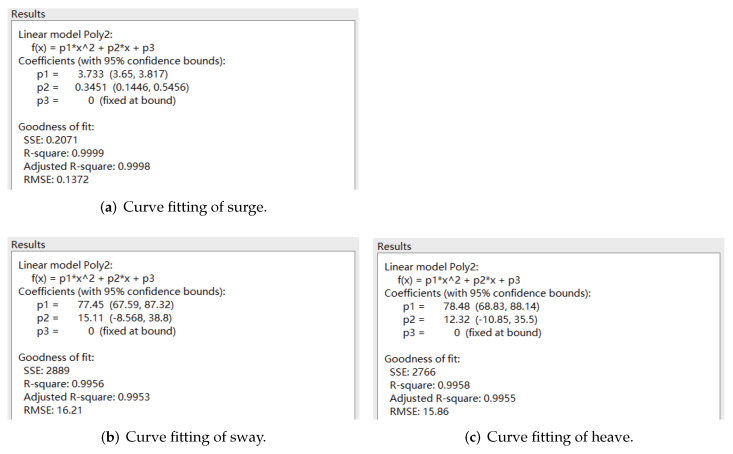
Curve fitting of uniform translational motion.

The velocity cloud diagrams of the AUV at a uniform velocity of 1 m/s in the surge, sway and heave directions are shown in Figure 5. Table 3 shows the calculated viscous drag on the AUV at different velocities. The least square method was used to fit the velocity values of translational motion and the corresponding viscous resistance data to a quadratic curve through the origin, and the fitting results are shown in Figure 6.

**Figure 7:**
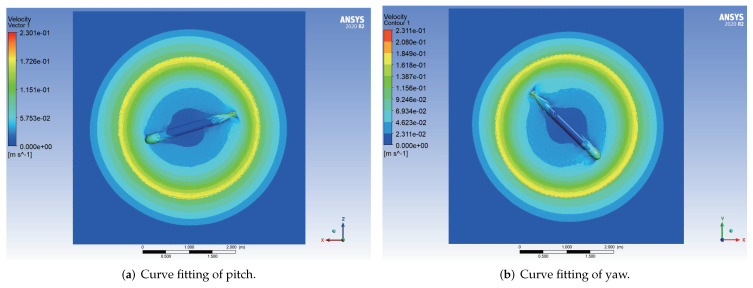
Velocity cloud diagrams of the AUV in uniform rotational motion.

**Figure 8:**
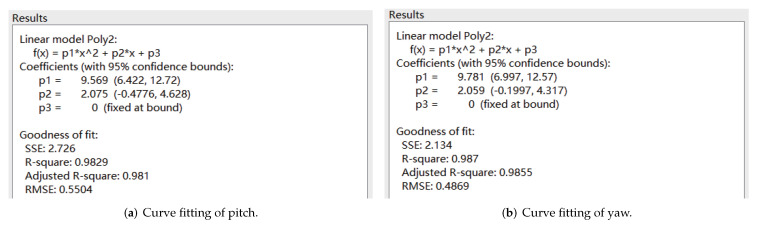
Curve fitting of uniform rotational motion.

The velocity cloud diagrams of the AUV moving around the *z* axis with a uniform rotational velocity of 0.5 rad/s are shown in Figure 7. The calculated damping moments of the AUV at different angular velocities are given in Table 4. The least square method was used to fit the rotational velocity values and the corresponding viscous moment data to a quadratic curve through the origin, and the fitting results are shown in Figure 8.

The approximate viscous hydrodynamic coefficients obtained by fitting the results are shown in Table 5.

According to Equation (7), it can be clearly seen that the inertial hydrodynamic force on the AUV has a linear relationship with its acceleration. The next simulation considers the uniform acceleration of the AUV to 2m/s at different linear accelerations and to 1rad/s at different angular accelerations, because the results obtained by solving for the state of the AUV at different accelerations contain the drag force and moment caused by the viscous hydrodynamic force. Therefore, the inertial hydrodynamic force due to the additional mass should be the difference between the results for the uniform-acceleration and uniform-velocity motion solutions. By fitting this difference to a linear relationship, the approximate inertial hydrodynamic coefficients can be obtained. The user-defined function (udf) is used to write programs to achieve uniform acceleration of the AUV translational motion and uniform acceleration of its rotational motion.

**Figure 9:**
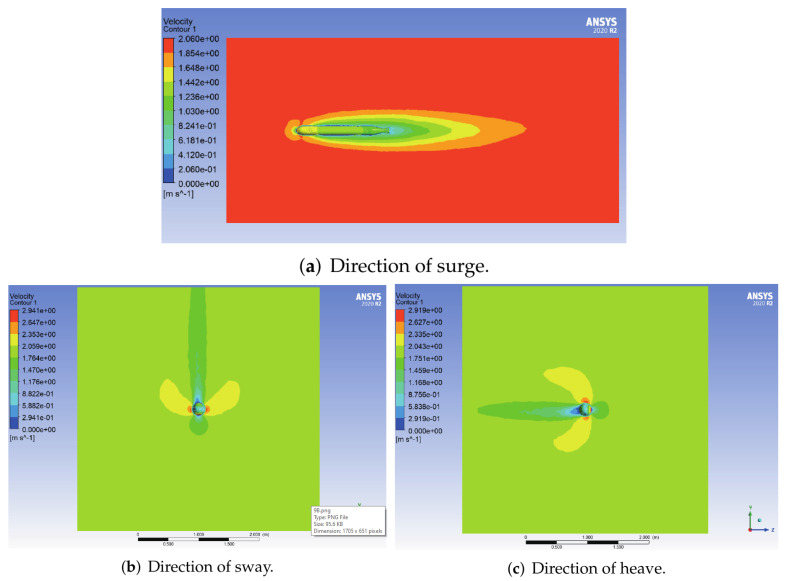
Velocity cloud diagrams of the AUV in uniformly accelerating translational motion.

**Figure 10:**
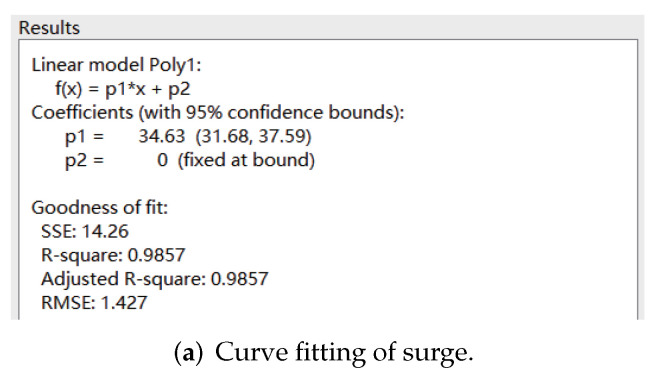

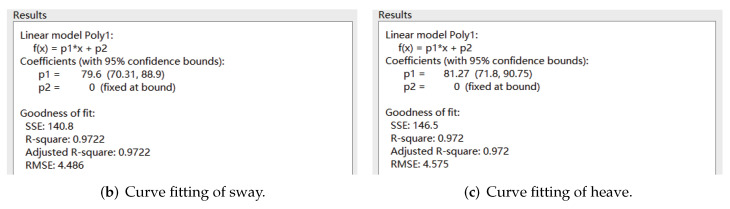
Curve fitting of uniform accelerating translational motion.

Figure 9 shows the velocity cloud diagrams of the AUV with a uniform acceleration of $0.1\;m/s^{2}$ in the surge, sway and heave directions. Table 6 shows the calculated results for the drag force on the AUV at different accelerations. The least square method was used to fit the acceleration values and drag data to a straight line through the origin, and the fitting results are shown in Figure 10.

**Figure 11:**
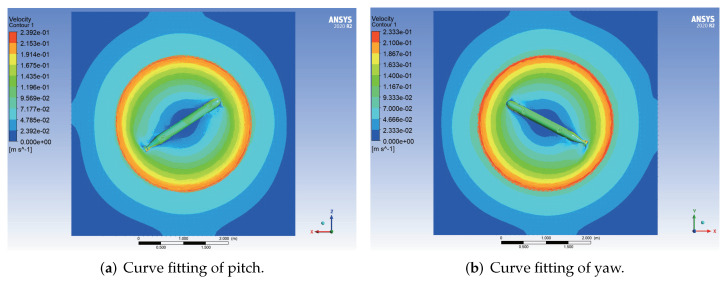
Velocity cloud diagrams of the AUV in uniformly accelerating rotational motion.

**Figure 12:**
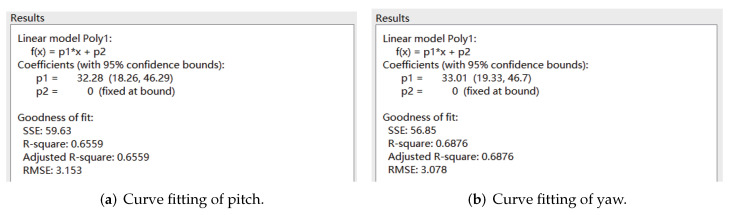
Curve fitting of uniform accelerating rotational motion.

The velocity cloud diagrams of the AUV in uniform rotational motion around the *z* axis with an angular acceleration of $0.01\;\mathrm{rad}/s^{2}$ are shown in Figure 11. The calculated results for the moments of the AUV at different angular accelerations are given in Table 7. The least square method was used to fit the angular acceleration values and moment data to a straight line through the origin, and the fitting results are shown in Figure 12.

The approximate inertial hydrodynamic coefficients obtained by fitting the results are given in Table 8.

## 4. Design of the Controller

**Figure 13:**
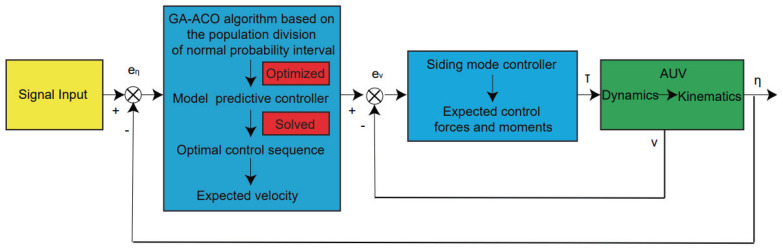
Diagram of the AUV control framework.

In this part, the AUV controller used to perform trajectory tracking tasks is designed in detail. The controller is composed of two cascaded controllers. The outer loop controller uses the novel GA-ACO algorithm based on the population division of the normal probability interval, combined with the constraints of velocity and position and the system output feedback, to solve the optimal control sequence of the model predictive controller, that is, the expected velocity command of the AUV, which is transmitted to the inner loop controller. The inner loop controller is a sliding mode controller that can obtain the control input instructions of the AUV and ensure tracking of the AUV along its entire trajectory. The control strategy block diagram is shown in Figure 13.

### 4.1. MPC Based on Novel GA-ACO

It is assumed that the trajectory is given in advance and is smooth and bounded. The expected trajectory is described as follows:

(8) $$Y_{d}={\left[x_{d},y_{d},z_{d},\theta_{d},\psi_{d}\right]}^{T}$$

The expected velocity command is obtained by inputting the error between the actual position of the AUV and the expected position. $T_{s}$ is the sampling time, and the motion model of the system is obtained after discretization as follows:

(9) $$\eta \left(k+1\right)=\eta \left(k\right)+J\left(k\right)v\left(k\right)T_{s}$$

To ensure stable motion of the AUV, the velocity increment is given as an input, and the state-space model of the AUV is expressed as follows:

(10) $$X\left(k+1\right)={\left[\eta \left(k+1\right),v\left(k\right)\right]}^{T}$$

The velocity increment is defined as follows:

(11) Δv(k)=v(k)−v(k−1)

Y(k) is defined as the output. By defining matrices $Q_{A}\left(k\right)$ and $Q_{B}\left(k\right)$ in the form given below, the equation of state can be rewritten as follows:

(12) $$X\left(k+1\right)=Q_{A}\left(k\right)X\left(k\right)+Q_{B}\left(k\right)\Delta v\left(k\right)$$

(13) Y(k)=CX(k)

with

(14) $$Q_{A}\left(k\right)=\left[\begin{array}{cc} I_{5} & J\left(k\right)T_{s}\\ 0_{5\times 5} & I_{5} \end{array}\right],Q_{B}\left(k\right)=\left[\begin{array}{c} J(k)T_{s}\\ I_{5} \end{array}\right],\;C=\left[\begin{array}{cc} I_{5} & 0_{5\times 5} \end{array}\right]$$

where $I_{5}$ is a 5×5 identity matrix and $0_{5}$ is a 5×5 zero matrix. Equations (10) and (11) are the model prediction equations for the AUV, from which the future state of the system can be inferred. The prediction horizon is denoted by $N_{p}$, and the control horizon is denoted by $N_{c}$; these horizons must satisfy $N_{c}\leq N_{p}$. The control value is held constant outside the control horizon. The controller output sequences are as follows:

(15) $$X_{N_{p}}=\left[\begin{array}{c} X(k+1|k)\\ X(k+2|k)\\ \vdots \\ X(k+N_{p}|k) \end{array}\right],Y_{N_{p}}=\left[\begin{array}{c} Y(k+1|k)\\ Y(k+2|k)\\ \vdots \\ Y(k+N_{p}|k) \end{array}\right]$$

The system input sequence is as follows:

(16) $$\Delta v\left(k\right)=\left[\begin{array}{c} \Delta v(k+1|k)\\ \Delta v(k+2|k)\\ \vdots \\ \Delta v(k+N_{p}-1|k) \end{array}\right]$$

Based on the principle of MPC, the model prediction equations can be further expressed as

(17) $$\bar{X}\left(k+1\right)={\bar{Q}}_{A}\left(k\right)\bar{X}\left(k\right)+{\bar{Q}}_{B}\left(k\right)\Delta v\left(k\right)$$

where

(18) $${\bar{Q}}_{A}\left(k\right)={\left[Q_{A}\left(k\right),Q_{A}{\left(k\right)}^{2},\cdots ,Q_{A}{\left(k\right)}^{N_{p}}\right]}^{T}$$

(19) $${\bar{Q}}_{B}\left(k\right)=\left[\begin{array}{cccc} Q_{B}\left(k\right) & 0 & \cdots & 0\\ Q_{A}\left(k\right)Q_{B}\left(k\right) & Q_{B}\left(k\right) & \cdots & 0\\ \vdots & \vdots & \ddots & \vdots \\ Q_{A}{\left(k\right)}^{N_{p}-1}Q_{B}\left(k\right) & Q_{A}{\left(k\right)}^{N_{p}-2}Q_{B}\left(k\right) & \cdots & Q_{B}\left(k\right) \end{array}\right]$$

Constraints: The upper and lower bounds on the AUV state and the velocity increment are set as follows:

(20) $$u_{\min}\leq \Delta v\left(k\right)\leq u_{\max}$$

(21) $$X_{\min}\leq X\left(k\right)\leq X_{\max}$$

By adjusting the current and future inputs to the system, the predicted performance cost function is defined as follows:

(22) $$E\left(t\right)=\int_{0}^{T}[∥Y\left(t\right)-Y_{d}{\left(t\right)∥}_{Q_{y}}^{2}+{∥\Delta v\left(t\right)∥}_{Q_{\Delta v}}^{2}]dt$$

where

(23) $${∥y∥}_{Q_{y}}^{2}=y^{T}Q_{y}y,{∥\Delta vs.∥}_{Q_{\Delta v}}^{2}=\Delta v^{T}Q_{\Delta v}\Delta v$$

$Q_{y}$ and $Q_{\Delta v}$ are symmetric positive-definite weight matrices. Within the sampling time $T_{s}$, the cost function E(t) is discretized as follows:

(24) $$E\left(k\right)=\sum_{i=1}^{N_{p}}[∥Y\left(k+i|k\right)-Y_{d}{\left(k+i|k\right)∥}_{Q_{y}}^{2}+{∥\Delta v\left(k+i-1|k\right)∥}_{Q_{\Delta v}}^{2}]$$

According to Equation (13), the above equation can be transformed into

(25) $$E\left(k\right)=\sum_{i=1}^{N_{p}}[∥X\left(k+i|k\right)-X_{d}{\left(k+i|k\right)∥}_{Q_{x}}^{2}+{∥\Delta v\left(k+i-1|k\right)∥}_{Q_{\Delta v}}^{2}]$$

where

(26) $$X\left(k+i\right)=C^{†}Y\left(k+i\right),Q_{x}=C^{†}Q_{y}C$$

$C^{†}$ is the pseudoinverse of the matrix *C*. According to Equation (17), the simplified equation can be expressed as follows:

(27) $$E\left(k\right)=∥\bar{X}\left(k\right)-{\bar{X}}_{d}{\left(k\right)∥}_{{\bar{Q}}_{x}}^{2}+{∥\Delta v\left(k\right)∥}_{{\bar{Q}}_{\Delta v}}^{2}$$

where

(28) $${\bar{Q}}_{x}=\left[\begin{array}{cccc} Q_{x} & 0 & \cdots & 0\\ 0 & Q_{x} & \cdots & 0\\ \vdots & \vdots & \ddots & \vdots \\ 0 & 0 & \cdots & Q_{x} \end{array}\right]$$

(29) $${\bar{Q}}_{\Delta v}=\left[\begin{array}{cccc} Q_{\Delta v} & 0 & \cdots & 0\\ 0 & Q_{\Delta v} & \cdots & 0\\ \vdots & \vdots & \ddots & \vdots \\ 0 & 0 & \cdots & Q_{\Delta v} \end{array}\right]$$

It can be seen from Equations (27)–(29) that E(k) is a function of Δv(k); thus, the optimization problem can be transformed into the following problem of finding the minimum value of the cost function with constraints (20) and (21):

(30) $$\Delta v^{*}\left(k\right)=\underset{\Delta v(k)}{\mathrm{argmin}}E\left(k\right)$$

### 4.2. Novel GA-ACO for Solving the Cost Function

In this section, we introduce a novel GA-ACO method to replace the QP method in the standard MPC to solve the extreme value of the cost function and obtain the optimal control variable Δv(k). The proposed population normal probability division method is helpful to maintain the diversity of the GA population, to avoid falling into the local optimal solution and to improve the global search ability of the algorithm. To improve the convergence speed in the later stage of the algorithm, the initial optimization result of the GA is used as the initial pheromone of the ACO algorithm, and the global optimal solution is obtained by the rapid convergence of the ACO algorithm.

**Figure 14:**
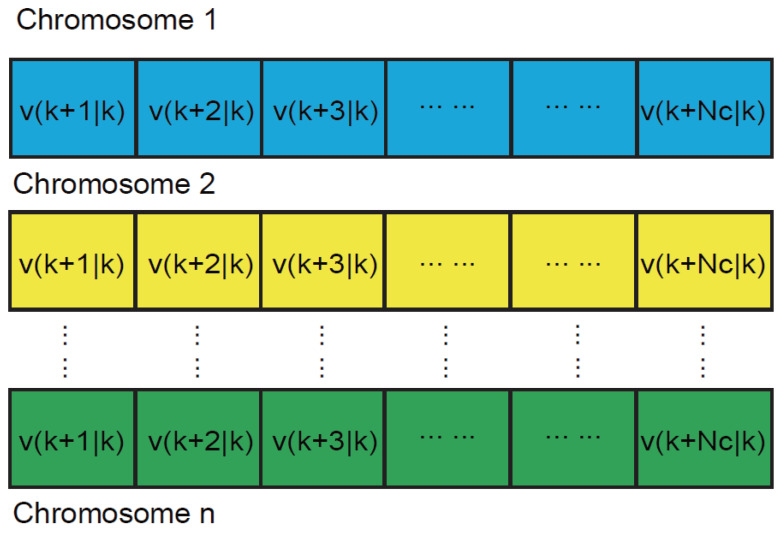
Chromosome coding.

**Figure 15:**
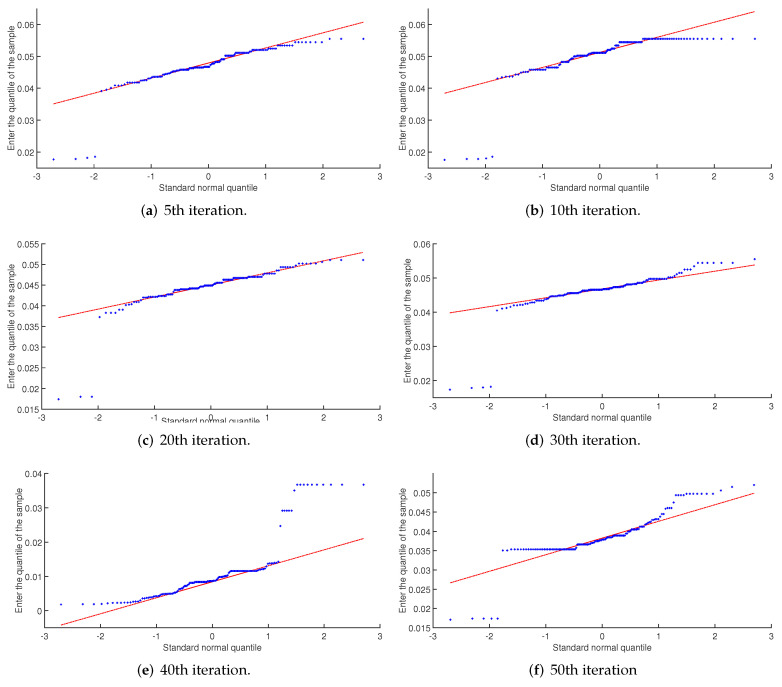
Results of the normal monitoring of population fitness values.

1. Chromosome coding and fitness function setting.
  Chromosomes are encoded in real numbers. As shown in Figure 14, the length of the chromosomes is consistent with the control horizon of MPC, where each locus corresponds to the predicted control amount in each sampling period of MPC. Taking Equation (27) as the fitness function of the algorithm, through continuous iteration, the optimal control sequence for each sampling period is obtained.
2. Population normal probability division.
  To reasonably improve the diversity of the population in the GA algorithm, a method of dividing the population normal probability interval is proposed. Equation (31) is a one-dimensional normal distribution function, with a probability density function given as Equation (32), where σ is the standard deviation and μ is the mean value. According to the 3σ criterion, the probability that the value is distributed in (μ−σ,μ+σ) is 68.26%, the probability of being distributed in (μ−2σ,μ+2σ) is 95.44%, and the probability of being distributed in (μ−3σ,μ+3σ) is 99.74%, which basically represents the entire probability space. The initial population is sorted according to the fitness value from greatest to least, and the population is divided into six families according to the above method. In this paper, the initial population number is set to 200, giving the division results shown in Table 9.
  (31) $$f\left(x\right)=\frac{1}{\sqrt{2\pi }\sigma }exp\left(-\frac{{\left(x-\mu \right)}^{2}}{2\sigma^{2}}\right)$$
  (32) $$\int_{a}^{b}\frac{1}{\sqrt{2\pi }\sigma }exp\left(-\frac{{\left(x-\mu \right)}^{2}}{2\sigma^{2}}\right)\;dx$$
3. Crossover, mutation and convergence.
  (1) Intra-family crossover: The optimal individual within the family is retained without a crossover operation. In the same family, individuals were randomly selected for intrafamily crossover, and the elite retention strategy was adopted. The parents and offspring were sorted according to the fitness function value, and the optimal retention was selected.
  (2) Cross between families: Set the maximum and minimum number of iterations, when the number of iterations is less than the maximum number of iterations, and update the optimum population fitness values that are either not changed or for which the rate of change is small. For the crossover operation between families, randomly select the best individual in the families of two different crossover operations, and take the elite reserved strategy after the cross. The idea of interfamily crossover controls the hemming distance of crossover individuals, avoids inbreeding, helps to maintain population diversity and prevents the algorithm from falling into a local minimum.
  (3) Mixed mutation operator: Hybrid mutation integrates the advantages of Gaussian mutation, Cauchy mutation, Levy mutation and single point mutation to make all of the mutation types work together. In the initial state, the selection probability of the four mutation operators is the same, where all are 0.25. The elite retention strategy is implemented after the mutation operation. If the offspring fitness of the individual after mutation is better than that of the parent, then the probability of the individual type occurring with the corresponding mutation strategy is increased; if the offspring fitness value of the individual is lower than that of the parent after mutation, then the probability of the individual type occurring in the corresponding mutation strategy is reduced. Finally, the probability of updating the four mutation modes after each population mutation operation is determined.
  (4) Algorithm convergence: In the proposed algorithm, by combining the advantages of the GA and ACO algorithms, the ant colony algorithm adopts the maximum-minimum ant algorithm. The preliminary optimal solution obtained by the GA is used as the initial pheromone of the ACO algorithm, allowing the algorithm to quickly converge to the global optimal control sequence $\Delta v^{*}\left(k\right)$, which makes up for the disadvantage of the slow convergence of the GA algorithm in the later stage. Take the first predicted value $\Delta v^{*}\left(k+1|k\right)$ of the optimal control sequence as the expected velocity control command.
4. Population normal probability judgment.
  Due to crossover and mutation within the population, it is necessary to monitor the normality of the population fitness value during the iteration process. It is proposed to use the absolute value difference of the median, mode and mean of the population fitness value of each iteration as the threshold for monitoring normality. According to the observations of several simulation results in the later period, the threshold value is set as 0.05. When the data normality quality is low and the minimum value of the difference value is greater than 0.05, the normal probability interval family is terminated. If convergence to the optimal solution has been achieved, then the optimal path is output and executed. Otherwise, the random crossover and mutation algorithm is adopted, and the elite retention strategy is implemented. As shown in Figure 15, using the simulation tool Quantile-Quantile Plots, the initial population number is 200, the maximum number of iterations is 50, and the 5th to 50th and the normality monitoring results of the population fitness value at the 50th generation. Excluding the special values, the results show that the sample points all fall near the red standard normal distribution line, which conforms to the normal probability distribution.

### 4.3. Dynamic Sliding Mode Velocity Controller

In this part, we design a dynamic sliding mode velocity controller. The conventional SMC control law is usually designed as a discontinuous function, which leads to chattering in the whole system. To overcome the chattering problem of SMC, we put the discontinuous function into the first derivative of the control input, so that the derivative of the control input is discontinuous, but the control input becomes a continuous function after integration, which can effectively suppress or eliminate the chattering problem of SMC.

The dynamic sliding mode controller is designed to overcome the external interference of THE AUV in the offshore environment and improve the robustness of the whole AUV control system. The force and torque required to drive the AUV are designed according to the input velocity error.

The difference between the expected velocity of the system according to Equation (33) and the actual velocity of the AUV is used as the input to the velocity controller. This error is defined as follows:

(33) $$e_{v}=v_{d}-v={\left[e_{u},e_{v},e_{w},e_{q},e_{r}\right]}^{T}$$

where $v_{d}={\left[v_{du},v_{dv},v_{dw},v_{dq},v_{dr}\right]}^{T}$. According to the velocity error $e_{v}$, the dynamic sliding surface is designed as follows:

(34) $$S=\delta ({\dot{e}}_{v}+e_{v})=0$$

where δ is the sliding surface coefficient. The following form can be obtained by deriving the sliding surface:

(35) $$\dot{S}=\delta \left({\ddot{e}}_{v}+{\dot{e}}_{v}\right)=\delta \left({\ddot{v}}_{d}-\ddot{v}\right)+\delta \left({\dot{v}}_{d}-\dot{v}\right)$$

We take the sliding mode surface s as the system state, design the sliding mode surface again as follows:

(36) $$\sigma =\dot{S}+S=\delta \left({\ddot{e}}_{v}+2{\dot{e}}_{v}+e_{v}\right)$$

The derivative of sliding mode surface in Equation (36) is obtained and is equal to the exponential asymptotic law:

(37) $$\dot{\sigma }=\ddot{S}+\dot{S}=\delta \left({\ddot{e}}_{v}+2{\dot{e}}_{v}+e_{v}\right)=-\sigma -sgn\sigma$$

Here, sgn is a discontinuous symbolic function, denotes the velocity at which the moving point of the system approaches the switching surface σ=0.

It can be concluded from Equations (36) and (37) that the discontinuous function causing SMC chattering only appears in sliding mode surface σ, that is, the discontinuous function only appears in the derivative of control input. After integrating the sliding mode surface σ, the control input is a continuous function, which eliminates the chattering problem of dynamic SMC. According to Equation (3), we obtain

(38) $$\dot{v}=M^{-1}\left(\tau -C\left(v\right)v-D\left(v\right)v-g\left(\eta \right)\right)$$

According to Equations (3)–(5), by combining Equations (34) and (35), the amount of control for each degree of freedom of the AUV can be obtained:

(39) $$\tau_{u}=\left(m-X_{\dot{u}}\right)({\dot{v}}_{du}-\frac{1}{2}\left(u-v_{du}-{\ddot{v}}_{du}+\ddot{u}-\frac{\int -\sigma -sgn\sigma d\sigma }{\delta }\right)+mw\left(q-r\right)-Z_{\dot{w}}wq-Y_{\dot{v}}vr+\left(X_{u}+X_{\left|u\right|u}\left|u\right|\right)u$$

(40) $$\tau_{v}=\left(m-Y_{\dot{v}}\right)({\dot{v}}_{dv}-\frac{1}{2}\left(v-v_{du}-{\ddot{v}}_{dv}+\ddot{v}-\frac{\int -\sigma -sgn\sigma d\sigma }{\delta }\right)+ur\left(m-X_{\dot{u}}\right)+\left(Y_{v}+Y_{\left|v\right|v}\left|v\right|\right)v$$

(41) $$\tau_{w}=\left(m-Z_{\dot{w}}\right)({\dot{v}}_{dw}-\frac{1}{2}\left(w-v_{dw}-{\ddot{v}}_{dw}+\ddot{w}-\frac{\int -\sigma -sgn\sigma d\sigma }{\delta }\right)-uq\left(m+X_{\dot{u}}\right)+\left(Z_{w}+Z_{\left|w\right|w}\left|w\right|\right)w$$

(42) $$\tau_{q}=\left(I_{y}-M_{\dot{q}}\right)({\dot{v}}_{dq}-\frac{1}{2}\left(q-v_{dq}-{\ddot{v}}_{dq}+\ddot{q}-\frac{\int -\sigma -sgn\sigma d\sigma }{\delta }\right)+uw\left(Z_{\dot{w}}-X_{\dot{u}}\right)+\left(M_{q}+M_{\left|r\right|q}\left|q\right|\right)q+bZ_{g}sin\left(\theta \right)$$

(43) $$\tau_{r}=\left(I_{z}-N_{\dot{r}}\right)({\dot{v}}_{dr}-\frac{1}{2}\left(r-v_{dr}-{\ddot{v}}_{dr}+\ddot{r}-\frac{\int -\sigma -sgn\sigma d\sigma }{\delta }\right)+uv\left(X_{\dot{u}}-Y_{\dot{v}}\right)+\left(N_{r}+N_{\left|r\right|r}\left|r\right|\right)r$$

At each sampling time *k*, $\Delta v^{*}\left(k\right)$ is recalculated. Then, the optimal control forces and moments are repeatedly computed and executed by the AUV to achieve rolling optimization. The optimization process iterates until the AUV completes the trajectory tracking task.

## 5. Stability Analysis

The stability of the controller proposed in this paper is analyzed in this section.

**Lemma** **1.** *The stability of the controller is proven using the traditional method of proving Lyapunov stability in control theory, i.e., by finding a Lyapunov function of the system that is positive definite and whose its derivative is negative definite.*

**Theorem** **1.** *Suppose that the following statements are true: (1) When k=0, the constrained optimization problem (30) has a solution Δv(k|k). (2) The zero state of the system output is measurable. For the nominal system (without considering external disturbances or system uncertainty), the following can be concluded: (a) For any k>0, the constrained optimization problem (30) updated with the state measurement X(k) has a solution. (b) The closed-loop system described by Equations (12) and (13) are nominally stable.*

**Lemma** **2.** *If the continuous differentiable function f(t), when t approaches infinity, has a finite limit value, and the derivative of f(t) is uniformly continuous, then*

$$\lim_{x\to +\infty }\left(\dot{f(t)}\right)=0$$

**Proof of Lemmas 1 and 2 and Theorem 1.**  Suppose that the solution to the optimization problem at time *k* is expressed as follows:

(44) $$\Delta v^{*}\left(k\right)=\left[\begin{array}{c} \Delta v^{*}\left(k|k\right)\\ \Delta v^{*}\left(k+1|k\right)\\ \vdots \\ \Delta v^{*}\left(k+N_{p}-1|k\right) \end{array}\right]$$

This approach satisfies the control constraints (20) and (21). The terminal constraint of the model predictive controller is set as follows:

(45) $$X(k+N_{p}|k)=0$$

The predicted optimal state sequence and output sequence are as follows:

(46) $$\left\{X_{k}^{*}\left(k+1|k\right),X_{k}^{*}\left(k+2|k\right),\cdots ,X_{k}^{*}\left(k+N_{p}-1|k\right),0\right\}\left\{Y_{k}^{*}\left(k+1|k\right),Y_{k}^{*}\left(k+2|k\right),\cdots ,Y_{k}^{*}\left(k+N_{p}-1|k\right),0\right\}$$

These sequences satisfy the output constraints (20) and (21) and the terminal constraint (45).

The optimal value of the cost function is:

(47) $$E^{*}\left(k\right)=\sum_{i=0}^{N_{p}-1}[∥X^{*}\left(k+i|k\right)-X_{d}{\left(k+i|k\right)∥}_{Q_{y}}^{2}+∥\Delta v^{*}\left(k+i|k\right){∥}_{Q_{\Delta v}}^{2}]$$

For the model predictive controller based on MPC-GA-ACO, the optimal cost function $E^{*}\left(k\right)$ is selected as the Lyapunov function $L_{m}^{*}\left(k\right)$:

(48) $$L_{m}^{*}\left(k\right)=\sum_{i=0}^{N_{p}-1}[∥X^{*}\left(k+i|k\right)-X_{d}{\left(k+i|k\right)∥}_{Q_{y}}^{2}+∥\Delta v^{*}\left(k+i|k\right){∥}_{Q_{\Delta v}}^{2}]$$

Clearly, Equation (48) satisfies $L_{m}^{*}\left(0\right)=0$ with k=0 and $L_{m}^{*}\left(k\right)>0$ with arbitrary k≠0.

In Equations (44) and (46), $X^{*}\left(k|k\right)=X\left(k\right)$ and $\Delta v^{*}\left(k|k\right)=\Delta v\left(k\right)$. By substituting into Equation (12), we obtain:

(49) $$X\left(k+1\right)=Q_{A}\left(k\right)X\left(k\right)+Q_{B}\left(k\right)\Delta v^{*}\left(k|k\right)=X^{*}\left(k+1\right)$$

At time k+1, a predictive control sequence is selected as follows:

(50) $$\Delta v\left(k+1\right)=\left[\begin{array}{c} \Delta v(k+1|k+1)\\ \Delta v(k+2|k+1)\\ \vdots \\ \Delta v(k+N_{p}-1|k+1)\\ \Delta v(k+N_{p}|k+1) \end{array}\right]=\left[\begin{array}{c} \Delta v^{*}\left(k+1|k\right)\\ \Delta v(k+2|k)\\ \vdots \\ \Delta v(k+N_{p}-1|k)\\ 0 \end{array}\right]$$

Due to the terminal constraint given in Equation (45), the last element of the preselected control sequence is zero, and the control constraint is satisfied. The corresponding state sequence is as follows:

(51) $$X\left(k+i+1|k+1\right)=\left\{\begin{array}{cc} X^{*}\left(k+i+1|k\right) & ,i=0,\cdots ,N_{p}-1\\ 0 & ,i=N_{p} \end{array}\right.$$

By substituting Equations (50) and (51) into Equation (25), we obtain:

(52) $$\begin{array}{cc} E(k+1) & =\sum_{i=0}^{N_{p}-1}[∥X\left(k+i+1|k+1\right)-X_{d}{\left(k+i+1|k+1\right)∥}_{Q_{x}}^{2}+{∥\Delta v\left(k+i+1|k+1\right)∥}_{Q_{\Delta v}}^{2}]\\ & =\sum_{i=0}^{N_{p}-2}[∥X^{*}\left(k+i+1|k\right)-X_{d}{\left(k+i+1|k\right)∥}_{Q_{x}}^{2}+∥\Delta v^{*}\left(k+i+1|k\right){∥}_{Q_{\Delta v}}^{2}]\\ & =\sum_{i=1}^{N_{p}-1}[∥X^{*}\left(k+i|k\right)-X_{d}{\left(k+i|k\right)∥}_{Q_{x}}^{2}+∥\Delta v^{*}\left(k+i|k\right){∥}_{Q_{\Delta v}}^{2}]\\ & =\sum_{i=0}^{N_{p}-1}[∥X^{*}\left(k+i|k\right)-X_{d}{\left(k+i|k\right)∥}_{Q_{x}}^{2}+∥\Delta v^{*}{\left(k+i|k\right)∥}_{Q_{\Delta v}}^{2}]-∥X^{*}\left(k|k\right)-X_{d}{\left(k|k\right)∥}_{Q_{x}}^{2}-{∥\Delta v^{*}\left(k|k\right)∥}_{Q_{\Delta v}}^{2}\\ & =E^{*}\left(k\right)-∥X^{*}\left(k|k\right)-X_{d}{\left(k|k\right)∥}_{Q_{x}}^{2}-{∥\Delta v^{*}\left(k|k\right)∥}_{Q_{\Delta v}}^{2} \end{array}$$

Obviously,

(53) $$E^{*}\left(k+1\right)\leq E\left(k+1\right)=E^{*}\left(k\right)-∥X^{*}\left(k|k\right)-X_{d}{\left(k|k\right)∥}_{Q_{x}}^{2}-{∥\Delta v^{*}\left(k|k\right)∥}_{Q_{\Delta v}}^{2}$$

(54) $$L_{m}^{*}\left(k+1\right)\leq L_{m}^{*}\left(k\right)$$

Equation (48) satisfies $L_{m}^{*}\left(0\right)=0$ with k=0 and $L_{m}^{*}\left(k\right)<0$ with arbitrary k≠0. The Lyapunov function in (46) is monotonically decreasing, i.e., $L_{m}^{*}\left(k+1\right)\leq L_{m}^{*}\left(k\right)$. Thus, system (17) is nominally stable.

For the dynamic sliding mode velocity controller, Since the expected trajectories of AUV designed in this paper are all functions of time *t*, the system is a nonautonomous system, and its stability can be proved according to Lemma 2. the Lyapunov function $L_{s}^{*}\left(k\right)$ is defined as follows:

(55) $$L_{s}^{*}\left(k\right)=\frac{1}{2}\sigma^{2}$$

(56) $${\dot{L}}_{s}^{*}\left(k\right)=\dot{\sigma }\sigma =\left(-\sigma -sgn\sigma \right)\sigma =-\sigma^{2}-\left|\sigma \right|$$

From Equations (55) and (56), It can be readily shown that $L_{s}^{*}\left(k\right)\geq 0$ and ${\dot{L}}_{s}^{*}\left(k\right)<0$. Since σ and $\dot{\sigma }$ are bounded, ${\ddot{L}}_{s}^{*}\left(k\right)$ is bounded and ${\dot{L}}_{s}^{*}\left(k\right)$ is uniformly continuous. From Lemma 2, we can get that when t approaches infinity, ${\dot{L}}_{s}^{*}\left(k\right),\sigma \;and\;S$ approaches zero. It can be obtained from Equations (34)–(36) that ${\dot{e}}_{v}=-e_{v}$. So we can get that as follows:

(57) $$e_{v}=\varepsilon e^{-t}$$

It can be obtained from Equations (57) that when time *t* approaches infinity, $e_{v}$ approaches zero, that is, the output of the system can track the expected signal, so the system is asymptotically stable. Lyapunov function $L_{s}^{*}\left(k\right)$ still satisfies Theorem 1, that is, $L_{s}^{*}\left(k\right)$ is positive definite and its derivative is negative definite. It can also prove the asymptotic stability of the dynamic sliding mode controller designed in this paper. The asymptotic stability of the inner loop controller and the outer loop controller ensures the overall stability of the AUV control system. □

## 6. Simulation Results

In this section, the effectiveness and robustness of the controller proposed in this paper (MPC-GA-ACO) are verified using the MATLAB/Simulink simulation platform, and the simulation results for the proposed controller are compared with those for a GA model predictive controller (MPC-GA) [35] and a standard model predictive controller (MPC) [38]. The above three controllers all form a cascade control with dynamic SMC. The main hydrodynamic parameters of the AUV used in the simulations are shown in Table 5 and Table 8.

The parameter settings for the controller proposed in this paper are as follows: The sampling period is Ts = 0.1 s. The prediction horizon is $N_{p}=10$, the control horizon is $N_{c}=6$, the weighted factors are $Q_{x}=diag\left(0.05,0.05,0.05,0.05,0.05\right)$ and $Q_{\Delta v}=diag\left(1,1,1,1,1\right)$, the amplitudes of the input constraints are $u_{\max}=[2,2,2,0.2,$${0.2]}^{T}$ and $u_{\min}={\left[-2,-2,-2,-0.2,-0.2\right]}^{T}$, and the state constraints are $X_{\max}=[+\infty ,+\infty ,$0.1,2π,2π,${2,2,2,\pi /6,\pi /6]}^{T}$ and $X_{\min}=[-\infty ,-\infty ,-0.1,-2\pi ,-2\pi ,-2,-2,-2,-\pi /6,$${-\pi /6]}^{T}$. In the novel MPC-GA-ACO, the number of populations is set to p=200, and the maximum number of iterations is 50. The sliding surface coefficient δ is set to 2.

Random disturbances are also introduced in the simulation as follows:

(58) $$\left\{\begin{array}{c} u_{r}=0.2\times \mathrm{rand}\left(1\right),\left(m/s\right)\\ v_{r}=0.2\times \mathrm{rand}\left(1\right),\left(m/s\right)\\ w_{r}=0.2\times \mathrm{rand}\left(1\right),\left(m/s\right) \end{array}\right.$$

where rand(1) is a normally distributed noise signal with mean 0 and variance 1.

The initial position and pose vector of the AUV is $\eta \left(0\right)={\left[0,5,0,0,0\right]}^{T}$. Expected trajectory 1 of the AUV in the simulation is as follows:

(59) $$\left\{\begin{array}{cc} x_{d} & =7\cos(0.1t)\\ y_{d} & =7\sin(0.1t)\\ z_{d} & =-0.2t \end{array}\right.$$

The expected trajectory 2 of the AUV in the simulation is as follows:

(60) $$\left\{\begin{array}{cc} x_{d} & =5\sin(0.2t)+10\sin(0.05t)\\ y_{d} & =5\sin(0.1t)+10\sin(0.025t)\\ z_{d} & =-0.1t \end{array}\right.$$

**Figure 16:**
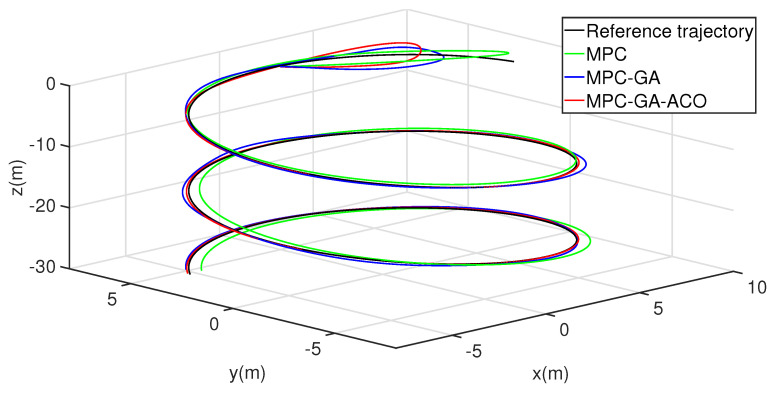
AUV trajectory 1 tracking without disturbance.

Figure 16 shows the trajectory 1 tracking performance of the AUV without disturbance. The simulation results show that the three controllers can give the AUV good trajectory tracking performance. Obviously, the controller designed in this paper is the best of the three controllers in the initial speed of tracking the expected trajectory and the final tracking performance (red curve).

**Figure 17:**
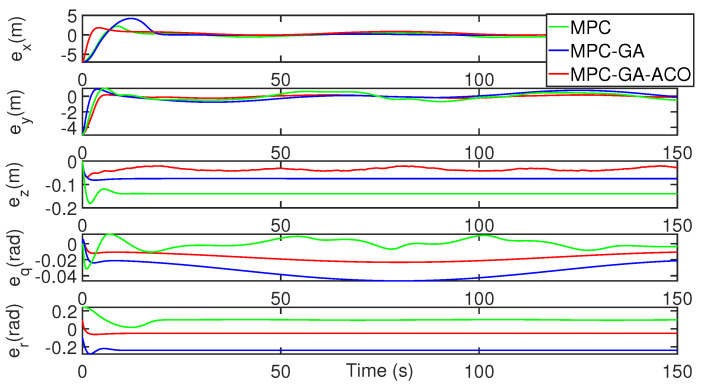
AUV trajectory 1 tracking error without disturbance.

Figure 17 shows a comparison of the tracking error results of each degree of freedom of the AUV and the tracking errors of the three control methods are bounded. Although the tracking error of the controller designed in this paper is lower than that of the standard model predictive controller in the initial stage, its overall tracking error result is better than that of the other two controllers (red curve). Table 10 shows the average position error of the three control methods in tracking the desired trajectory. It can be clearly seen from the table that the average error of the controller designed in this paper in five degrees of freedom is the smallest. The average position error data of surge, sway, heave, pitch and yaw are, respectively, 0.1431 m, 0.1654 m, 0.0999 m, 0.1178 m, 0.0987 m.

**Figure 18:**
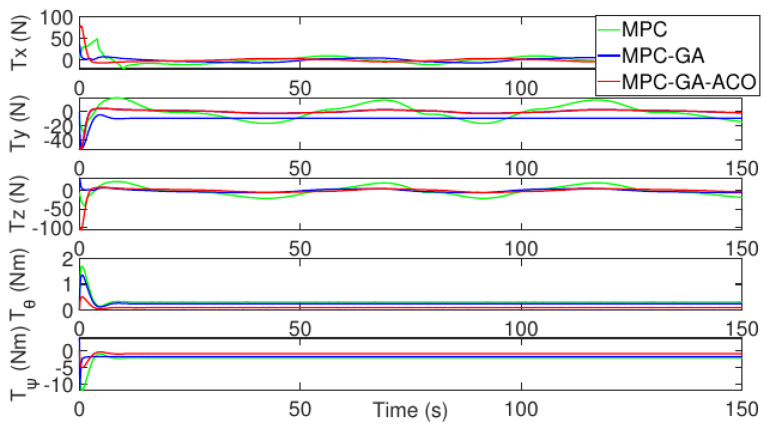
Control input of AUV without disturbance for trajectory 1.

As shown in Figure 18, the control input stability of the controller proposed in this paper is the best in the comparison of the control input of the three controllers without disturbance (red curve).

**Figure 19:**
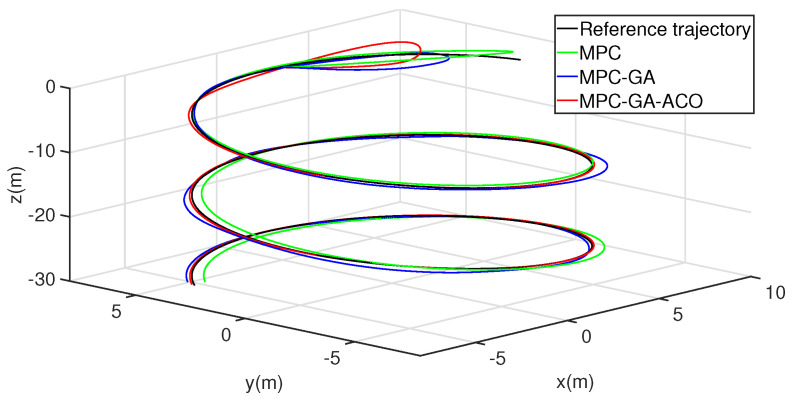
AUV trajectory 1 tracking with random disturbance.

Figure 19 shows the simulation results of trajectory 1 tracking performance with the random disturbance. Compared with the simulation results without disturbance, the random disturbance interferes with the motion of AUV in the three degrees of freedom of surge, sway and heave. Obviously, the tracking performance of the three control methods is slightly reduced. However, compared with the other two controllers, the controller proposed in this paper still has the best tracking performance (red curve).

**Figure 20:**
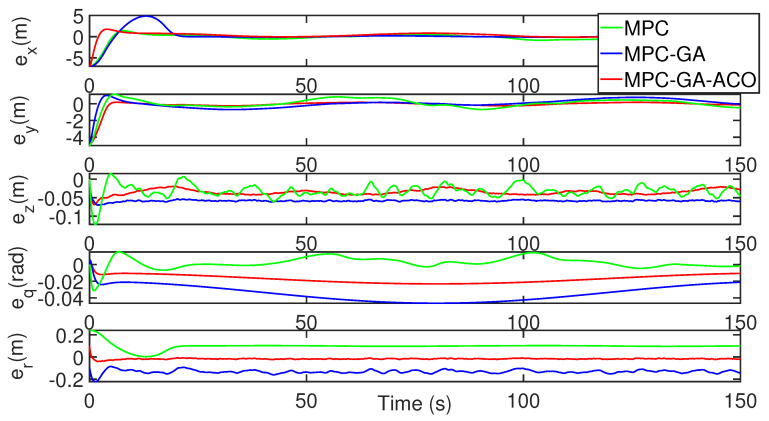
AUV trajectory 1 tracking error with random disturbance.

Figure 20 shows the comparison of the tracking error results of each degree of freedom of AUV, and the tracking error of the three control methods is bounded. The error fluctuates with the random disturbance, and the error fluctuation of standard MPC controller is the largest (green curve). However, the controller proposed in this paper has the minimum tracking error and the error fluctuation is stable (red curve). Table 11 shows the average position error of the three control methods when tracking the expected trajectory. It can be clearly seen from the table that the average position error of the controller designed in this paper is the smallest under five degrees of freedom. The average position error data of surge, sway, heave, pitch and yaw are 0.1893 m, 0.1867 m, 0.1445 m, 0.1498 m and 0.1612 m, respectively.

**Figure 21:**
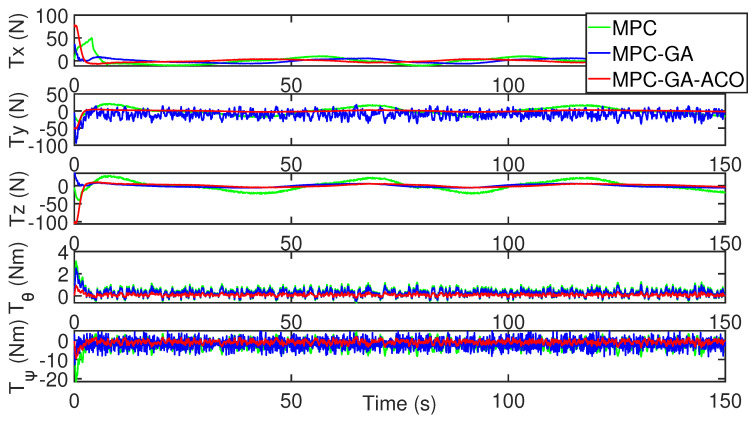
Control input of AUV with random disturbance for trajectory 1.

Figure 21 shows the control input fluctuation of the three controllers in the case of random disturbance. The control input stability of the proposed controller is still the best among the controllers and the fluctuation is the smallest (red curve).

**Figure 22:**
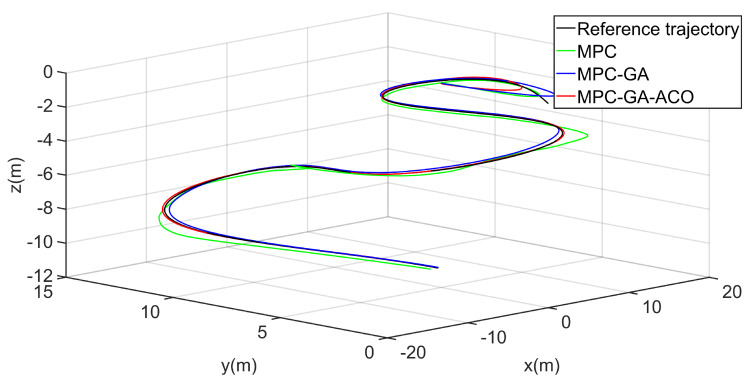
AUV trajectory 2 tracking without disturbance.

To further verify the performance of the controller, the trajectory tracking the performance of AUV is simulated again in aperiodic function trajectory 2. Figure 22 shows the trajectory 2 tracking performance of AUV without disturbance. The overall tracking effect of the standard MPC controller is poor (green curve). Although the overall tracking effect of the MPC-GA controller is improved compared with the standard MPC controller, the tracking speed is the slowest in the initial stage of trajectory tracking (blue curve). Because the new GA-ACO algorithm can reasonably maintain the diversity of the population, and ensure the local search and global search ability of the algorithm at the same time, so that the optimal control sequence can be obtained in each sampling period of MPC, the simulation results show that the controller proposed in this paper not only ensures the tracking speed of AUV in the initial stage of trajectory tracking but also the overall tracking effect is the best of the three (red curve).

**Figure 23:**
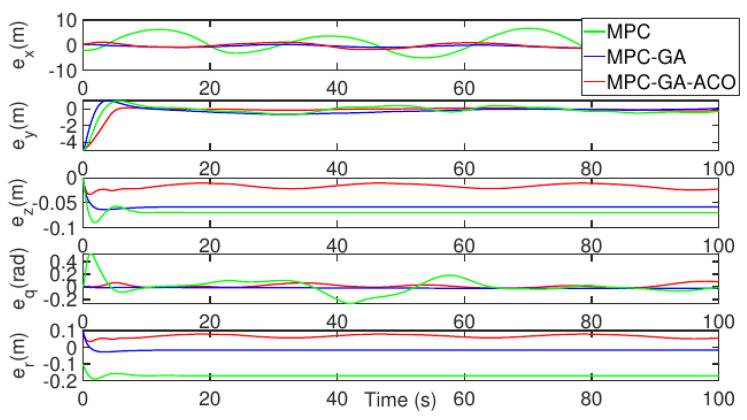
AUV trajectory 2 tracking error without disturbance.

Figure 23 shows the comparison of the tracking error results of each degree of freedom of AUV, and the tracking errors of the three control methods are bounded. Among them, the standard MPC controller error fluctuates greatly when the AUV bow direction change rate is large (green curve), but the controller proposed in this paper has the smallest tracking error (red curve). Table 12 shows the average position error of the three control methods when tracking the desired trajectory. It can be seen from the table that the average error of the controller designed in this paper is the smallest under five degrees of freedom. The average position error data of surge, sway, heave, pitch and yaw are 0.1881 m, 0.1825 m, 0.1110 m, 0.0915 m, and 0.0873 m, respectively.

**Figure 24:**
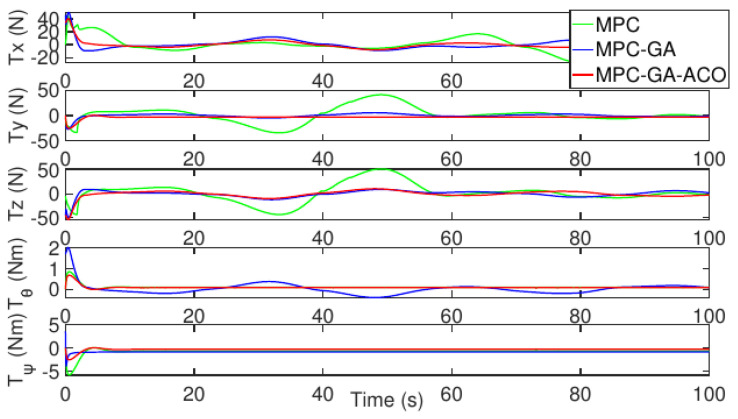
Control input of AUV without disturbance for trajectory 2.

Figure 24 shows the control input fluctuation of the three controllers without disturbance. The control input stability of the proposed controller is still the best and the fluctuation is the smallest (red curve).

**Figure 25:**
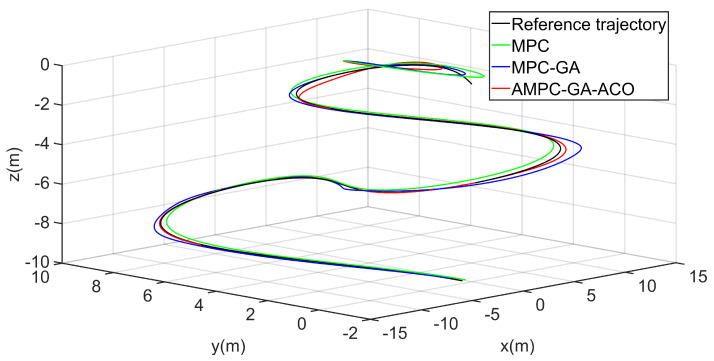
AUV trajectory 2 tracking with random disturbance.

**Figure 26:**
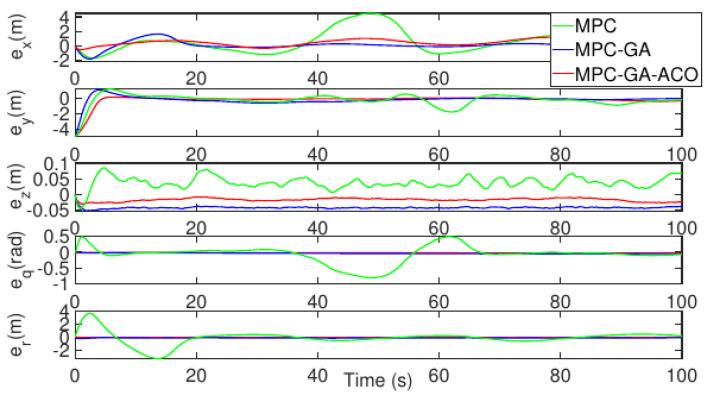
AUV trajectory 2 tracking error with random disturbance.

**Figure 27:**
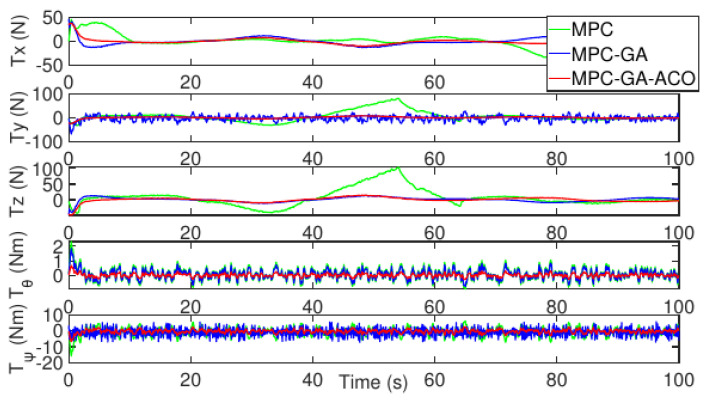
Control input of AUV with random disturbance for trajectory 2.

Figure 25, Figure 26 and Figure 27 show the trajectory 2 tracking performance of the AUV with random disturbance. Compared with the other two controllers, the controller achieves the optimal trajectory tracking performance under the condition of minimum error and smooth control intput under random disturbance. The random disturbance leads to a high-frequency variation of the desired speed command, but the proposed controller can still track the desired speed perfectly, which proves its effectiveness and robustness (red curve).

Table 13 shows the average position error of the three control methods in tracking the desired trajectory. It can be clearly seen from the table that the average error of the controller designed in this paper in five degrees of freedom is the smallest. The average position error data of surge, sway, heave, pitch and yaw are, respectively, 0.1932 m, 0.1894 m, 0.1777 m, 0.0998 m, 0.1221 m.

## 7. Conclusions

In this paper, a novel AUV double closed-loop trajectory tracking method based on model prediction and sliding mode cascade control is designed. First, a kinematics analysis of the fully driven AUV is carried out, and the kinematics and dynamics equations of five degrees of freedom are established. Second, the CFD software ANSYS Fluent is used to simulate the uniform velocity and uniform acceleration of AUV in surge, sway, heave, pitch and yaw degrees of freedom, and solve the main hydrodynamic coefficients required to establish the dynamic model. Then, a novel GA-ACO algorithm based on normal probability interval population division is proposed is proposed. Combined with the constraints of actual control input and state, the algorithm is applied to the outer loop MPC to solve the optimal velocity control sequence, generate the expected velocity command, which is passed to the inner loop. Compared with standard MPC and MPC-GA, this method can reasonably increase the diversity of GA population, improve the local and global search ability of the algorithm, and avoid the problem of falling into local optimal solution caused by QP or GA. Then combined with ACO algorithm, the convergence speed of the algorithm can be accelerated and the global optimal speed control sequence can be obtained for each sampling period. The inner loop uses dynamic SMC to generate the available control input of AUV to ensure the closed-loop tracking along the whole trajectory. A rolling time-domain implementation can be used to recalculate the optimal control input at each sampling instant, compensating for system uncertainties caused by modeling and external disturbances. Finally, two different types of reference desired trajectories are simulated to verify the effectiveness and robustness of the proposed controller under uncertain disturbances in the simulated marine environment. It is improved in reducing the position error of trajectory tracking and increasing the input stability of the controller. In the future research, we will further solve the adverse impact of SMC chattering on the controller, and strengthen the analysis of the impact of the actual marine environment on the control disturbance of AUV and the research on practical problems such as thruster output saturation.

## Figures and Tables

**Table 1:** AUV parameters.

Parameter	Value	Parameter	Value
Mass	84.615 kg	Diameter	0.3 m
Length	1.85 m	$I_{y}$; $I_{z}$	24.235 kgm^2^

**Table 2:** Values of ANSYS fluent parameters.

**Parameter of Translational Motion**	**Value**
Fluid area size	8.5m×4m×4m
Overall grid control	Curvature control function
Growth rate	1.2
Curvature normal angle	$18^{\circ }$
Number of mesh nodes	747,048
Number of mesh elements	4,112,607
**Parameter of Rotational Motion**	**Value**
Fluid area size	5m×5m×5m
Rotation area size	Diameter: 3m Height: 5m
Overall grid control	Curvature control function
Growth rate	1.2
Curvature normal angle	$18^{\circ }$
Number of mesh nodes	698,224
Number of mesh elements	3,348,218

**Table 3:** Data for uniform translational motion.

Velocity (m/s)	Force (*x*) (N)	Force (*y*) (N)	Force (*z*) (N)
0.1	0.0537	0.8773	0.8727
0.2	0.1081	5.6044	5.7074
0.3	0.4263	7.8390	7.9125
0.4	0.8867	16.5096	16.3312
0.5	1.0583	21.8900	22.0917
0.7	2.2160	42.3060	41.9060
1.0	4.0096	83.9660	84.0191
1.3	7.0015	138.3200	138.1212
1.5	8.8106	189.7001	182.4700
2.0	15.3730	388.4032	385.8816
2.5	24.2423	510.7725	511.2045
3.0	34.6884	734.2678	735.6437

**Table 4:** Data for uniform rotational motion.

Angular Velocity (Rad/s)	Moment (*y*) (N·m)	Moment (*z*) (N·m)
0.1	0.3456	0.3563
0.2	0.5907	0.6809
0.3	1.5908	1.6358
0.4	2.5004	2.5417
0.5	3.1156	3.0756
0.6	5.2103	5.1967
0.7	6.6678	6.7621
0.8	7.2111	7.3007
0.9	8.6017	8.9889
1.0	12.4451	12.5146

**Table 5:** Viscous hydrodynamic coefficients.

Parameter	Value
$X_{u}$	0.35 kg/s
$Y_{v}$	15.11 kg/s
$Z_{w}$	12.32 kg/s
$M_{q}$	2.08 ${\mathrm{kgm}}^{2}/\left(s\cdot \mathrm{rad}\right)$
$N_{r}$	2.06 ${\mathrm{kgm}}^{2}/\left(s\cdot \mathrm{rad}\right)$
$X_{\left|u\right|u}$	3.73 kg/s
$Y_{\left|v\right|v}$	77.45 kg/s
$Z_{\left|w\right|w}$	78.48 kg/s
$M_{\left|q\right|q}$	9.57 ${\mathrm{kgm}}^{2}/{\mathrm{rad}}^{2}$
$N_{\left|r\right|r}$	9.78 ${\mathrm{kgm}}^{2}/{\mathrm{rad}}^{2}$

**Table 6:** Data for uniformly accelerating translational motion.

Acceleration (m/s^2^)	Force (*x*) (N)	Force (*y*) (N)	Force (*z*) (N)
0.01	0.0606	0.8654	0.8359
0.02	0.3561	1.8364	2.4069
0.05	0.9167	6.5482	6.9586
0.1	5.9878	11.8842	12.9393
0.2	8.5703	19.3711	19.9455
0.5	18.9163	48.5111	48.9941
1.0	33.3043	74.0327	75.7163

**Table 7:** Data for uniformly accelerating rotational motion.

Angular Acceleration (m/s^2^)	Moment (*y*) (N·m)	Moment (*z*) (N·m)
0.01	1.2292	1.1818
0.02	1.4877	1.4067
0.05	2.2315	2.1993
0.1	8.4114	8.3320
0.2	11.1191	11.2121
0.5	13.1224	13.5543

**Table 8:** Inertial hydrodynamic coefficients.

Parameter	Value
$X_{\dot{u}}$	34.63 kg/s
$Y_{\dot{v}}$	79.60 kg/s
$Z_{\dot{w}}$	81.27 kg/s
$M_{\dot{q}}$	32.28 ${\mathrm{kgm}}^{2}/\left(s\cdot \mathrm{rad}\right)$
$N_{\dot{r}}$	33.01 ${\mathrm{kgm}}^{2}/\left(s\cdot \mathrm{rad}\right)$

**Table 9:** Population interval division.

Interval	1	2	3	4	5	6
Probability families	(−3σ,−2σ)	(−2σ,−σ)	(−σ,0)	(0,σ)	(σ,2σ)	(2σ,3σ)
Probability	2.5%	13.5%	34%	34%	13.5%	2.5%
Population number	5	27	68	68	27	5

**Table 10:** Average position error without disturbance in trajectory 1.

Methods (m/s)	*x* (m)	*y* (m)	*z* (m)	θ (rad)	ψ (rad)
MPC	0.2005	0.1932	0.2343	0.1257	0.1282
MPC-GA	0.2011	0.1881	0.1881	0.1331	0.1016
MPC-GA-ACO	0.1431	0.1654	0.0999	0.1178	0.0987

**Table 11:** Average position error with random disturbance in trajectory 1.

Methods (m/s)	*x* (m)	*y* (m)	*z* (m)	θ (rad)	ψ (rad)
MPC	0.3011	0.2352	0.1901	0.1859	0.2225
MPC-GA	0.2459	0.2237	0.1725	0.1811	0.1783
MPC-GA-ACO	0.1893	0.1867	0.1445	0.1498	0.1612

**Table 12:** Average position error without disturbance in trajectory 2.

Methods (m/s)	*x* (m)	*y* (m)	*z* (m)	θ (rad)	ψ (rad)
MPC	0.3066	0.2961	0.2005	0.2517	0.2011
MPC-GA	0.3312	0.2192	0.1834	0.1551	0.1812
MPC-GA-ACO	0.1881	0.1825	0.1110	0.0915	0.0873

**Table 13:** Average position error with random disturbance in trajectory 2.

Methods (m/s)	*x* (m)	*y* (m)	*z* (m)	θ (rad)	ψ (rad)
MPC	0.3211	0.2714	0.2456	0.2117	0.2019
MPC-GA	0.3029	0.2321	0.1764	0.1770	0.2231
MPC-GA-ACO	0.1932	0.1894	0.1777	0.0998	0.1221

## Data Availability

Not applicable.
